# First Report of* bla*_CTX-M-28_ in Enterobacteriaceae Isolates in the United Arab Emirates

**DOI:** 10.1155/2018/1304793

**Published:** 2018-01-18

**Authors:** Mubarak Alfaresi, Garwin Kim Sing, Abiola Senok

**Affiliations:** ^1^College of Medicine, University of Sharjah, Sharjah, UAE; ^2^Department of Microbiology and Immunology, College of Medicine, Alfaisal University, Riyadh, Saudi Arabia; ^3^Department of Basic Medical Sciences, College of Medicine, Mohammed Bin Rashid University of Medicine and Health Sciences, Dubai, UAE

## Abstract

**Background:**

The CTX-M family of extended-spectrum beta lactamase (ESBL) enzymes is comprised of over 60 *bla*_CTX-M_ gene variants with the predominance of *bla*_CTX-M-15_ in many regions. In this report, we present the first description of *bla*_CTX-M-28_ in the United Arab Emirates.

**Methods:**

Forty-five non-duplicate ESBL producing isolates identified in a secondary care facility in the United Arab Emirates from June to July 2016 were studied. Gene sequencing was performed and DNA sequences were annotated using the BLAST program to identify the gene subtypes.

**Results:**

The majority of the ESBL positive isolates were* E. coli* (*n*/*N* = 39/45; 86.6%) followed by* K. pneumoniae* (*n* = 5) and* K. oxytoca* (*n* = 1). All isolates harboured *bla*_CTX-M_ and *bla*_TEM_ genes, 18 had *bla*_SHV_, and 2 were *bla*_VIM_ positive. Thirty-seven isolates (82.2%) were positive for *bla*_CTX-M-28_. Other *bla*_CTX-M_ genes identified include *bla*_CTX-M-167_ (*n* = 2; isolates #1 and 26) and one each for *bla*_CTX-M-38_, *bla*_CTX-M-163_, and *bla*_CTX-M-198_. No *bla*_CTX-M-15_ was identified. The predominant *bla*_TEM_ subtype was *bla*_TEM-171_ (*n* = 8) followed by one of each of *bla*_TEM-120_, *bla*_TEM-163_, and *bla*_TEM-206_. The *bla*_SHV_ subtypes were *bla*_SHV-148_ and *bla*_SHV-187_.

**Conclusion:**

The findings indicate the first description of *bla*_CTX-M-28_ in a setting where *bla*_CTX-M-15_ was previously predominant.

## 1. Introduction

Extended-spectrum beta lactamase (ESBL) producing Enterobacteriaceae are major agents of nosocomial and community acquired infections. In contrast to ESBL enzymes such as TEM, SHV, and OXA types which arose from point mutations, the CTX-M family of enzymes are derived from* Kluyvera* spp. chromosomal beta lactamase genes. Since the first description in 1992, the CTX-M family has now become the predominant ESBL enzymes in Enterobacteriaceae [1, 2]. The CTX-M family comprises over 60 *bla*_CTX-M_ variants which have been classified into five major phylogenetic clusters [3]. The rapid dissemination of CTX-M-positive ESBL producing bacteria has hitherto been largely driven by the predominance of CTX-M-15 across geographical regions and in diverse bacterial isolates [2]. The *bla*_CTX-M-28_ gene is closely related to the *bla*_CTX-M-15_ gene, differing only by a single amino acid at the C-terminus, and both belong to CTX-M-Group 1. In the Arabian Gulf region, one of the highest rates of ESBL prevalence has been from the United Arab Emirates (UAE) [4]. Previously reported work indicated a predominance of *bla*_CTX-M-15_ [5, 6]. In this study, we present the first report of *bla*_CTX-M-28_ in the UAE and its predominance among the Enterobacteriaceae isolates studied.

## 2. Methods

*Bacterial Isolates*. The study was carried out at a secondary care general hospital in the UAE. A total of 45 nonduplicate, consecutive, phenotypically confirmed ESBL isolates identified in the Microbiology Laboratory from June-July 2016 were studied. Only the first isolate per patient was included in the study. Isolates were obtained from clinical specimens submitted for routine diagnostic investigation. Bacterial identification and antibiotic susceptibility testing were carried out using the Vitek2 automated system (BioMerieux, Marcy-l'Étoile, France) and the N204 VITEK susceptibility card was used for ESBL confirmation. Isolates were stored at −80°C for molecular characterization. Ethical approval was obtained from hospital review board (Medical Executive Committee).

### 2.1. Genotypic Testing for ESBL

PCR was performed using previously described primers and protocol [7]. Each 25 *μ*L reaction mixture was made up of 12.5 *μ*L of GoTaq Green Master Mix (Promega Co., Madison, WI), 0.4 *μ*M primer, and 3 *μ*L of sample DNA. The PCR protocol followed was initial denaturation at 95°C for 5 min, cyclic denaturation at 95°C for 50 s, annealing at 56°C, 50°C, or 60°C for 40 s, elongation at 72°C for 1 min for 35 cycles, and final extension at 72°C for 5 min. Detection of PCR products was on 1% agarose gel. Positive amplicons were purified by Promega Wizard SV Gel and PCR Clean-up System (Promega Co., Madison, WI) and sequenced at Macrogen Laboratories (Seoul, South Korea). DNA sequences were annotated by using the BLAST program (https://blast.ncbi.nlm.nih.gov) to identify the gene subtypes. Briefly, raw AB1 sequence trace files were translated into FASTQ format and individual sequences quality-trimmed. Pairwise alignments of quality-filtered paired forward and reverse reads ≥200 nt were produced. Reference sequences of complete bacterial* bla* and* mcr-1* genes were retrieved from the NCBI nucleotide database. A reference BLAST nucleotide database was compiled from gene features (FASTA format) with makeblastdb v2.6.0+. F. The FASTA sequences were queried against the* bla*/*mcr-1* subtypes BLAST database. The query hits were checked for gene specificity (all hits had to be from the same gene to be valid). Sequences with a single highest bit score were assigned to the respective gene subtype. Query sequences with multiple highest bit scores were assigned to the respective gene type without specification of a subtype.

## 3. Results

Of the 208 Enterobacteriaceae identified during the study period, 45 (21.6%) were ESBL positive. The majority of the ESBL positive isolates were* E. coli* (*n*/*N* = 39/45; 86.6%) followed by* K. pneumoniae* (*n* = 5) and* K. oxytoca* (*n* = 1). Most were from inpatients (*n*/*N* = 34/45; 75.5%). The isolates were obtained from urine (*n* = 36), sputum (*n* = 4), pus (*n* = 4), and blood (*n* = 1). All the isolates harboured *bla*_CTX-M_ and *bla*_TEM_ genes, 18 had *bla*_SHV_ gene, and 2 were positive for *bla*_VIM_ gene.  Table 1 shows the distribution of the isolates and the ESBL genes identified in each isolate. Over half of the isolates (*n* = 25) harboured a combination of three genes and 19 had two genes (Table 1). One isolate harboured a combination of four genes, namely, *bla*_CTX-M-167_; *bla*_CTX-M-28_; *bla*_SHV_; *bla*_TEM_ (isolate #26, Table 1). Thirty-seven isolates (82.2%) were positive for *bla*_CTX-M-28_. Other *bla*_CTX-M_ genes identified include *bla*_CTX-M-167_ (*n* = 2; isolates #1 & 26), The other CTX-M subtypes identified include *bla*_CTX-M-167_ (*n* = 2) and one each for *bla*_CTX-M-38_, *bla*_CTX-M-163_, and *bla*_CTX-M-198_. With the exception of the *bla*_CTX-M-163_ isolate, all others were also positive for *bla*_CTX-M-28_. No *bla*_CTX-M-15_ was identified. For seven isolates (#9, 14, 22, 25, 28, 38, and 45, see Table 1), the CTX-M subtype could not be resolved as query sequences with multiple highest hit scores were identified.

The two *bla*_SHV_ subtypes identified were *bla*_SHV-148_ (isolate #4) and *bla*_SHV-187_ (isolate #8). The predominant *bla*_TEM_ subtype was *bla*_TEM-171_ (isolates #10, 13, 20, 28, 30, 32, 39, and 40, Table 1). The other *bla*_TEM_ subtypes were *bla*_TEM-120_ (isolate #42), *bla*_TEM-163_ (isolate #41), and *bla*_TEM-206_ (isolate #31). A total of 18 isolates were identified as harbouring *bla*_SHV_ but assignment into subtypes could not be done because query sequences with multiple highest hit scores were identified. Similarly, there were 33 isolates identified with *bla*_TEM_ but without subtype classification. Two isolates harboured *bla*_VIM_ (isolates #44 and 45). None of the isolates harboured the *bla*_OXA-48_, *bla*_IMP_, *bla*_NDM_, or* mcr-1* genes. The antibiotic susceptibility profile of each isolate is shown in the supplementary file (available here). There was no evidence of carbapenem resistance as all isolates were sensitive to imipenem, meropenem, and ertapenem. In addition, fosfomycin sensitivity was seen in all isolates; 18 were sensitive to ciprofloxacin and trimethoprim-sulphamethoxazole.

## 4. Discussion

During the study period 21.6% of Enterobacteriaceae identified in this healthcare facility were ESBL positive which is lower than the 36–41% previously reported in studies from the UAE [4, 5]. However, these previous studies were carried out over a one-year period and in multiple care facilities. This represents the first study from the UAE carried out in a single secondary care facility where cases coming straight from the community are seen. Thus the finding of a relatively high ESBL prevalence suggests that ESBL dissemination remains an ongoing occurrence in the community in the UAE. This is particularly pertinent as the majority of the isolates were obtained from urine.

Our findings identified several CTX-M, SHV, and TEM genes which had not been previously reported in the UAE. This is largely because of the sequencing approach which was used in this study. The predominant ESBL gene identified was *bla*_CTX-M_ which is in keeping with the previously reported work from the UAE and neighboring countries [5, 6, 8–10]. Indeed, CTX-M group one genes and specifically *bla*_CTX-M-15_ had been identified as the predominant CTX-M type in ESBL producers in the Arabian Gulf region [5, 10–12]. Previous work done in the UAE by Alfaresi et al. identified *bla*_CTX-M-15_ in majority of ESBL isolates [5]. The findings in this study indicate a predominance of *bla*_CTX-M-28_. Unlike *bla*_CTX-M-15_ which had been frequently been described in the literature, *bla*_CTX-M-28_ has been sparsely documented. Sporadic identification has been documented in reports from India, Brazil, Bosnia and Herzegovina, China, and Tunisia [13–18]. In the Arabian Gulf region, *bla*_CTX-M-28_ has only been identified in one* E. coli* isolate in Kuwait [19]. In contrast to these sporadic reports, 62.1% of the ESBL producers reported in South Korea by Yoo et al. were *bla*_CTX-M-28_ which was a shift from the predominance of *bla*_CTX-M-15_ [17]. The findings from this study represent the first report of similar predominance of *bla*_CTX-M-28_ in the Arabian Gulf region. Similar to the findings in South Korea, the majority of our isolates were* E. coli* [17]. Both *bla*_CTX-M-28_ and *bla*_CTX-M-15_ belong to CTX-M group 1 and differ by just a single amino acid at the 3′-end of the CTX-M gene. It has been suggested that because of this close similarity and the proximity of the sequence differences to the 5′-end (position 21) and 3′-end (position 865) of the CTX-M gene, the primers designed for detection of the CTX-M-1 group might misidentify these two CTX-M genes. This might explain why *bla*_CTX-M-28_ has hitherto been underreported. Previous work done by Alfaresi et al. [5] using a sequencing methodology similar to that used in the present study found predominance of *bla*_CTX-M-15_. Additionally, the primers used in this study were designed to ensure adequate differentiation of these two CTX-M genes taking into cognizance the recommendations by Menezes et al. [20]. Hence, these findings are therefore indicative of the first report of *bla*_CTX-M-28_ in the UAE. It was interesting to find that the two isolates harbouring the *bla*_VIM_ gene were carbapenem sensitive. It is possible that this might be due to nonexpression of this metallo-beta-lactamase gene and further work is needed to confirm this.

A limitation of our work is the short study duration and number of isolates reported. However, the findings indicate the need for multicenter studies to determine if there is an emerging shift in ESBL epidemiology and the clinical relevance of ESBL genes which had not been previously reported in our setting but were found in this study. Furthermore, such large studies will enable further understanding of the evolutionary trend and clinical implications of *bla*_CTX-M-28_. As *bla*_CTX-M-15_ was notorious for rapid dissemination globally, close surveillance and monitoring is needed for early detection of the spread of *bla*_CTX-M-28_.

## Figures and Tables

**Table 1:** Distribution of isolates and ESBL genes.

#	Collection date	Isolate	Site	Source	ESBL genes
(1)	6/16/2016	*E. coli*	Outpatient	Urine	*bla*_CTX-M-167_; *bla*_CTX-M-28_; *bla*_TEM_
(2)	6/16/2016	*E. coli*	Outpatient	Urine	*bla*_CTX-M-28_; *bla*_TEM_
(3)	6/19/2016	*E. coli*	Inpatient	Urine	*bla*_CTX-M-28_; *bla*_TEM_
(4)	6/19/2016	*E. coli*	Inpatient	Urine	*bla*_CTX-M-28_; *bla*_SHV-148_; *bla*_TEM_
(5)	6/19/2016	*K. pneumoniae*	Outpatient	Urine	*bla*_CTX-M-28_; *bla*_SHV_; *bla*_TEM_
(6)	6/25/2014	*E. coli*	Inpatient	Urine	*bla*_CTX-M-28_; *bla*_SHV-187_; *bla*_TEM_
(7)	6/26/2016	*E. coli*	Inpatient	Urine	*bla*_CTX-M-28_; *bla*_SHV_; *bla*_TEM_
(8)	6/26/2016	*E. coli*	Inpatient	Urine	*bla*_CTX-M-28_; *bla*_TEM_
(9)	6/29/2016	*E. coli*	Inpatient	Sputum	*bla*_CTX-M_; *bla*_SHV_; *bla*_TEM_
(10)	6/29/2016	*E. coli*	Inpatient	Blood	*bla*_CTX-M-28_; *bla*_TEM-171_
(11)	6/29/2016	*E. coli*	Inpatient	Urine	*bla*_CTX-M-28_; *bla*_TEM_
(12)	6/30/2016	*K. pneumoniae*	Inpatient	Urine	*bla*_CTX-M-28_; *bla*_SHV_; *bla*_TEM_
(13)	6/30/2016	*E. coli*	Inpatient	Urine	*bla*_CTX-M-28_; *bla*_SHV_; *bla*_TEM-171_
(14)	6/30/2016	*E. coli*	Inpatient	Urine	*bla*_CTX-M_; *bla*_SHV_; *bla*_TEM_
(15)	7/4/2016	*E. coli*	Inpatient	Urine	*bla*_CTX-M28_; *bla*_SHV_; *bla*_TEM_
(16)	7/5/2016	*K oxytoca*	Inpatient	Urine	*bla*_CTX-M-28_; *bla*_SHV_; *bla*_TEM_
(17)	7/5/2016	*E. coli*	Inpatient	Urine	*bla*_CTX-M-28_; *bla*_SHV_; *bla*_TEM_
(18)	7/6/2016	*E. coli*	Inpatient	Urine	*bla*_CTX-M-28_; *bla*_TEM_
(19)	7/6/2016	*E. coli*	Inpatient	Urine	*bla*_CTX-M-28_; *bla*_TEM_
(20)	7/8/2016	*E. coli*	Inpatient	Urine	*bla*_CTX-M-28_; *bla*_TEM-171_
(21)	7/8/2016	*E. coli*	Inpatient	Urine	*bla*_CTX-M-28_; *bla*_TEM_
(22)	7/10/2016	*E. coli*	NR	Sputum	*bla*_CTX-M_; *bla*_SHV_; *bla*_TEM_
(23)	7/11/2016	*E. coli*	Inpatient	Pus	*bla*_CTX-M-198_; *bla*_CTX-M-28_; *bla*_TEM_
(24)	7/12/2016	*E. coli*	Inpatient	Urine	*bla*_CTX-M-28_; *bla*_TEM_
(25)	7/12/2016	*K. pneumoniae*	Outpatient	Urine	*bla*_CTX-M_; *bla*_SHV_; *bla*_TEM_
(26)	7/12/2016	*E. coli*	Outpatient	Urine	*bla*_CTX-M-167_; *bla*_CTX-M-28_; *bla*_SHV_; *bla*_TEM_
(27)	7/13/2016	*E. coli*	Inpatient	Urine	*bla*_CTX-M-28_; *bla*_TEM_
(28)	7/13/2016	*E. coli*	Inpatient	Urine	*bla*_CTX-M_; *bla*_TEM-171_
(29)	7/13/2016	*K. pneumoniae*	Inpatient	Pus	*bla*_CTX-M-28_; *bla*_SHV_; *bla*_TEM_
(30)	7/14/2016	*E. coli*	Outpatient	Urine	*bla*_CTX-M-28_; *bla*_TEM-171_
(31)	7/15/2016	*E. coli*	Inpatient	Urine	*bla*_CTX-M-163_; *bla*_TEM-206_
(32)	7/18/2016	*E. coli*	Inpatient	Urine	*bla*_CTX-M-28_; *bla*_TEM-171_
(33)	7/18/2016	*E. coli*	Inpatient	Sputum	*bla*_CTX-M-28_; *bla*_SHV_; *bla*_TEM_
(34)	7/19/2016	*E. coli*	Outpatient	Urine	*bla*_CTX-M-28_; *bla*_SHV_; *bla*_TEM_
(35)	7/23/2016	*E. coli*	Outpatient	Urine	*bla*_CTX-M-28_; *bla*_CTX-M-38_; *bla*_TEM_
(36)	7/23/2016	*E. coli*	Inpatient	Urine	*bla*_CTX-M-28_; *bla*_TEM_
(37)	7/24/2016	*K. pneumoniae*	Inpatient	Urine	*bla*_CTX-M-28_; *bla*_TEM_
(38)	7/25/2016	*E. coli*	Inpatient	Pus	*bla*_CTX-M_; *bla*_TEM-206_
(39)	7/25/2016	*E. coli*	Inpatient	Sputum	*bla*_CTX-M-28_; *bla*_TEM-171_
(40)	7/25/2016	*E. coli*	Inpatient	Urine	*bla*_CTX-M-28_; *bla*_TEM-171_
(41)	7/26/2016	*E. coli*	Inpatient	Urine	*bla*_CTX-M-28_; *bla*_SHV_; *bla*_TEM-163_
(42)	7/26/2016	*E. coli*	Inpatient	Urine	*bla*_CTX-M-28_; *bla*_SHV_; *bla*_TEM-120_
(43)	7/27/2016	*E. coli*	Inpatient	Urine	*bla*_CTX-M-28_; *bla*_SHV_; *bla*_TEM_
(44)	7/30/2016	*E. coli*	Outpatient	Urine	*bla*_CTX-M-28_; *bla*_TEM_; *bla*_VIM_
(45)	7/30/2017	*E. coli*	Outpatient	Pus	*bla*_CTX-M_; *bla*_TEM_; *bla*_VIM_

*Notes*. NR: not recorded.

## Supplementary Materials

Supplementary Materials Specimen collection date/source/hospital unit and Antibiotic susceptibility profile for each isolate.
